# Sex and Species Differences in the Development of Diet-Induced Obesity and Metabolic Disturbances in Rodents

**DOI:** 10.3389/fnut.2022.828522

**Published:** 2022-02-17

**Authors:** Ivana Maric, Jean-Philippe Krieger, Pauline van der Velden, Stina Börchers, Mohammed Asker, Milica Vujicic, Ingrid Wernstedt Asterholm, Karolina P. Skibicka

**Affiliations:** ^1^Institute for Neuroscience and Physiology, University of Gothenburg, Gothenburg, Sweden; ^2^Wallenberg Centre for Molecular and Translational Medicine, University of Gothenburg, Gothenburg, Sweden; ^3^Department of Nutritional Sciences, Pennsylvania State University, University Park, PA, United States

**Keywords:** diet-induced obesity (DIO), brown adipose tissue (BAT), species differences, rats, mice, sex differences

## Abstract

Prevalence and health consequences of obesity differ between men and women. Yet, most preclinical studies investigating the etiology of obesity have, to date, been conducted in male rodents. Notably, diet is a major determinant of obesity, but sex differences in rodent models of diet-induced obesity, and the mechanisms that underlie such differences, are still understudied. Here, we aim to determine whether time course and characteristics of diet-induced obesity differ between sexes in rats and mice, and to investigate the potential causes of the observed divergence. To achieve this, we offered the most commonly tested rodents of both sexes, SD rats and C57BL/6 mice, a free choice of 60 % high-fat diet (HFD) and regular chow; body weight, food intake, fat mass, brown adipose responses, locomotor activity and glucose tolerance were assessed in a similar manner in both species. Our results indicate that overall diet-induced hyperphagia is greater in males but that females display a higher preference for the HFD, irrespective of species. Female rats, compared to males, showed a delay in diet-induced weight gain and less metabolic complications. Although male rats increased brown adipose tissue thermogenesis in response to the HFD challenge, this was not sufficient to counteract increased adiposity. In contrast to rats, female and male mice presented with a dramatic adiposity and impaired glucose tolerance, and a decreased energy expenditure. Female mice showed a 5-fold increase in visceral fat, compared to 2-fold increase seen in male mice. Overall, we found that male and female rodents responded very differently to HFD challenge, and engaged different compensatory energy expenditure mechanisms. In addition, these sex differences are divergent in rats and mice. We conclude that SD rats have a better face validity for the lower prevalence of overweight in women, while C57BL/6 mice may better model the increased prevalence of morbid obesity in women.

## Introduction

Obesity and related comorbidities are continuing to increase at an alarming rate. Modern lifestyle promotes eating that exceeds metabolic need, resulting in excessive weight gain (1). To investigate how exposure to palatable food promotes hyperphagia, adiposity, and disturbances in glucose homeostasis, rodent models of diet-induced obesity (DIO) have been widely used in obesity research. A broad range of energy-dense diets have been employed to induce weight gain and its adverse metabolic effects in rodents, all of them characterized by high sugar and fat content. Most commonly, commercially available diets with fat content >40% are used. Another less frequent choice is the use of a buffet of diets, when animals are offered a variety of human food items (cafeteria diet) or an addition of specific fat or sugar solutions.

Prevalence and clinical manifestations of overweight, obesity, and eating disorders differ between men and women (2–4). While more men than women are considered to be overweight, epidemiological studies show that women have an increased risk of developing obesity and morbid obesity (3). Fat distribution also clearly differs by sex; pre-menopausal women store more fat in subcutaneous depots, whereas men tend to present with a visceral adiposity that is associated with greater cardio-metabolic risks. Correspondingly, according to CDC, less women than men present with co-morbidities linked to an excessive adiposity, like diabetes and cardiovascular disease (5, 6). The increased susceptibility to obesity in women, along with the sexually divergent presentation of metabolic consequences, strongly suggest that the physiological response to an obesogenic environment differs by sex and calls for an investigation of these differences in animal models. Yet, most preclinical studies have been done in male rodents (7, 8), resulting in significant knowledge gaps in the female biology of energy homeostasis, and likely missed therapeutic opportunities.

Surprisingly, sex comparisons of diet-induced obesity in the most commonly used animal model species, mice, and rats, result in conflicting findings. Female rats are reported to be more vulnerable in response to a metabolic challenge (9–12). In contrast, studies in mice predominantly suggest that females are equally or less susceptible to dietary obesity, unless additionally challenged by age or ovariectomy (13–16). Male rats and mice eat more than respective females, yet the mechanisms or meal patterns contributing to this sex difference in intake may be species divergent in that male rats tend to eat larger meals but male mice eat same-size meals as female mice, just more frequently (17–19). Sex not only impacts the intake component of energy balance, but also energy expenditure. In comparison to white adipose tissue (WAT) which stores energy, brown adipose tissue (BAT) can dissipate energy through heat-generation (20, 21). However, the underrepresentation of female rodents in studies evaluating BAT function, results in overall poor understanding of BAT function in health and obesity in females, and how these processes might differ between sexes (22, 23).

The lack of metabolic studies concurrently including both males and females prevents accurate evaluation of how development of dietary obesity is interacting with sex. In addition, inconsistency in study designs with a variety of diet types, onset of intervention, and duration of diet, makes it challenging to elucidate what variables cause the observed sex differences, and which model best corresponds to sex differences seen in human obesity. Furthermore, inconsistencies might also come from the fact that two distinct rodent species are classically used for the evaluation of dietary obesity. Sprague Dawley rats and C57BL/6 mice are two of the most commonly used rodent strains in biomedical research. A major difference between the two, is that the first is an outbred strain and the latter inbred, giving rise to a divergent genetic and phenotypic variability. To the best of our knowledge, this is the first study that in a systematic manner evaluates DIO in both sexes of both species.

Thus, the main aim of this study was to determine whether time course and characteristics of diet-induced obesity differ between both sexes of rats and mice, maintained in the same animal facility and on the same diet combination, and to investigate the potential energy balance disturbances underlying the observed divergence.

## Methods And Materials

### Animals

Ten-week-old male and female Sprague-Dawley rats and C57BL/6N mice were fed regular chow (Envigo, 2018 Teklad Global 18% Protein Rodent Diet, 3.1 kcal/g) or a choice of chow and high-fat diet (Research Diets Inc., 60% energy from fat, 20% energy from both protein and carbohydrates, D12492, 5.21 kcal/g). The palatable diet utilized in the present study has a high content of lard, rich in saturated fatty acids, and sucrose. High dietary fat content has been positively correlated with body weight gain in both human and animal studies, whereas saturated fatty acids are shown to be more obesogenic than polyunsaturated fatty acids. While this type of diet is traditionally offered as a single diet, we used a free choice paradigm to better model the heterogenous human food environment. Food intake and body weight were measured biweekly during the first 10 weeks of the diet exposure. All data were collected during the light cycle. The terminal experiment took place after 14 and 16 weeks on the diet, for rats and mice, respectively. Animals were fasted for 4 h, decapitated under isoflurane anesthesia, and adipose tissues were flash frozen in liquid nitrogen and stored in −80°C after collection and weighing. Rats were pair-housed, whereas mice were housed in groups of ten, as required by Ethical permit. All rodents were housed in different rooms of the same facility at 21–22°C and 55–65% humidity under a 12-h light/dark cycle with *ad libitum* access to food and water, unless otherwise specified. All experiments were performed during the light phase. All studies were approved by the Animal Welfare Committee of the University of Gothenburg, Sweden, Ethical permit # 137/15.

### Temperature Measurements (FLIR)

BAT- and core temperature were assessed by imaging the surface body temperature of the interscapular area and flank, using infrared thermography (FLIR T500-Series thermal camera, with FLIR tools software) (24). The rat measurements were conducted at the beginning of the experiment, and during weeks 5 and 10 after introducing the high-fat diet. In mice, images were obtained after 10 weeks on the diet. The interscapular area and a 2 cm^2^ patch of the lumbar area were shaved 1 day before measurements to optimize temperature detection by removing the confounding effect of piloerection. Three images were taken of each animal in their home cage, and the average temperature was used for analysis.

### RNA Isolation and mRNA Expression

Brown, gonadal and inguinal adipose tissues pads were collected immediately after sacrifice performed after a brief isoflurane anesthesia, they were then weighed and frozen in liquid nitrogen. The white adipose tissue depots were collected unilaterally. Total RNA was extracted from BAT using RNeasy Lipid Tissue Mini kit (Qiagen), RNA quality and quantity were assessed spectrophotometrically by Nanodrop 1000 (NanoDrop Technologies). cDNA was synthesized using *iScript* cDNA Synthesis kit (Bio-Rad). Gene expression of genes involved in thermogenic control were measured (*n* = 9 per group) with real-time RT-PCR using the following TaqMan assays for rat tissues: Dio2-Rn00581867_m1, Adrb3-Rn00565393_m1, Ucp1-Rn00562126_m1, Gapdh-Rn01775763_g1, and mouse tissues: Dio2-Mm00515664_m1, Adrb3-Mm02601819_g1, Ucp1-Mm01244861_g1, Gapdh-Mm99999915_g1 (Applied Biosystems). Gene expression values were calculated based on the ΔΔCt method (25).

### Oral Glucose Tolerance Test

After 12 weeks of HFD exposure, an oral glucose tolerance test was performed to assess whole body glucose clearance. Rodents fasted for 4 h received an oral bolus of a glucose solution of 40% (2.5 g/kg, solved in PBS) (*n* = 8 per group for rats, *n* = 20 per group for mice). Blood samples were obtained from a tail vein nick immediately before and 15, 30, 60, 90, and 120 min after the oral gavage. Blood glucose levels were measured with the Bayer Contour XT (Bayer).

### Locomotor Activity

Locomotor activity was assessed after 11 weeks on the diet, by recording animals that were allowed to freely explore a lit square arena (100 cm × 100 cm × 30 cm) for 30 min (*n* = 20 per group, in both rats and mice) starting 3 h into the light cycle. Movement tracking was performed with the EthoVision (Noldus IT) tracking system. The animals were acclimatized to the room for a minimum of 30 min. The activity arena, however, represented a novel environment, thus the activity measured here portrays novelty-induced locomotion rather than home-cage spontaneous activity.

### Statistical Analysis

Data are presented as mean ± standard error of the mean (SEM). All statistical analyses and graphs were generated using GraphPad Prism 9. Analyses were conducted using Student's *t*-test for comparisons of two groups, or two-way ANOVA with *post-hoc* Holm-Sidak tests when appropriate. Statistical analysis combining both species is presented in Supplementary Table S1.

Linear regression was applied to test for a relationship between body weight and brown adipose tissue temperature. *p*-values lower than 0.05 were considered statistically significant.

## Results

### Body Weight and Food Intake Control in Response to a High-Fat Choice Diet Challenge Is Sex Divergent in Rats

**Figure 1:**
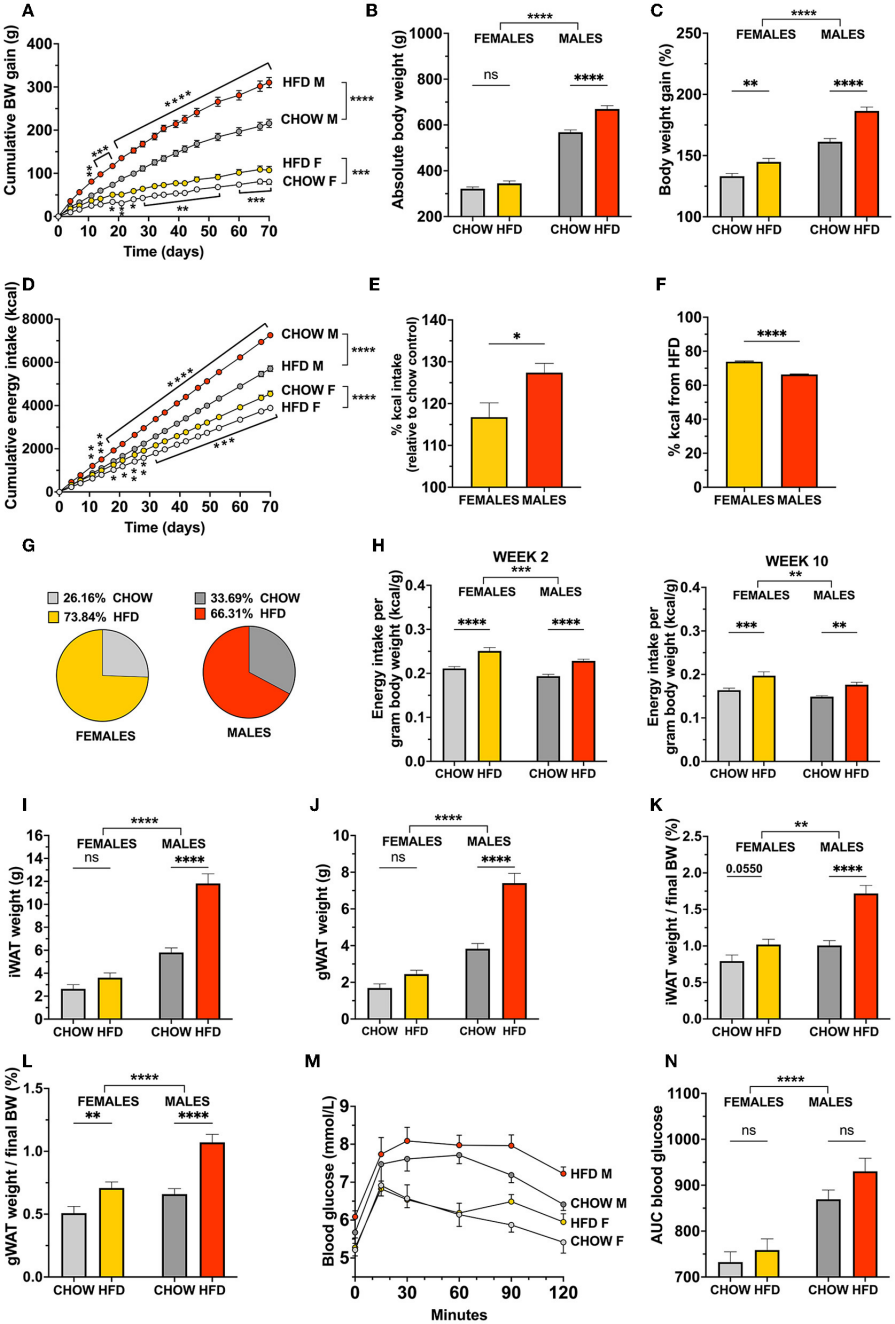
Body weight and food intake control in response to a high-fat choice diet challenge is sex divergent in rats. Offering an obesogenic choice diet to both female and male rats increased cumulative body weight **(A)** and promoted hyperphagia **(D)** compared to their respective chow-fed controls. Females chose to consume a higher proportion of their daily intake from HFD **(F,G)** but males displayed a greater level of total overeating **(E)**. Both sexes consumed more calories per body weight after 2 weeks, and 10 weeks on the high-fat diet **(H)**; left and right, respectively. Absolute body weight was significantly higher in high-fat fed males **(B)** but not females, unless analyzed as percent relative to starting weight **(C)**. Likewise, HFD led to increased fat mass in gonadal and inguinal adipose tissues in males only when absolute fat weight is analyzed **(I,J)**. When fat weight is expressed as % of body weight a trend to increased iWAT mass is detected in females and a significantly larger increase is found in males **(K)**. For gWAT while both sexes showed increased fat mass, the diet response was more pronounced in males **(L)**. Glucose tolerance was not impaired by the diet in either sex; however, male rats presented higher blood glucose levels across all conditions **(M,N)**. Data are expressed as mean ± SEM. *p < 0.05, **p < 0.01, ***p < 0.001, ****p < 0.0001 compared with respective controls, in **(A,D)**, the pairwise comparisons indicate the comparison between diet groups within each sex. *n* = 40 (females, 20 in each diet group); *n* = 39 (males, 19 in HFD group). OGTT: *n* = 9 per group. BW, body weight; HFD, high-fat diet; IWAT, inguinal (subcutaneous) white adipose tissue; GWAT, gonadal white adipose tissue; AUC, area under the curve.

In order to determine if the time course and presentation of diet-induced obesity is sex divergent in rats fed a high-fat choice diet (HFD), food intake and body weight of males and females was measured over 10 weeks. A significant increase in food intake [*F*_(18, 324)_ = 71.54, *p* < 0.0001] and body weight gain [*F*_(18, 666)_ = 26.32, *p* < 0.0001] was detected in males after 11 days (two-way ANOVA, Holm-Sidak's multiple comparisons test, *p* = 0.0027 and *p* = 0.0052 for cumulative kcal intake and body weight gain, respectively; Figures 1A,D) whereas females did not reach a consistent hyperphagia and weight accumulation until the following week (*p* < 0.05 and *p* < 0.05 for cumulative kcal intake and body weight gain on day 18; Figures 1A,D).

Interestingly, female rats did not show a significant change in body weight compared to controls (Figure 1B), unless assessed as percentage relative to starting weight (*p* = 0.0036, Figure 1C). In addition to the more rapid onset of overeating, male rats also showed a greater level of overconsumption throughout the entire intervention, calculated by comparing the average energy intake of the high-fat group with the average intake of the controls at the same timepoint (*p* = 0.0207, Figure 1E). Females on the other hand, chose to consume a higher proportion of their daily intake from HFD (*p* < 0.0001, Figures 1F,G). Two-way repeated measures ANOVA revealed a significant difference between males and females in both the main effect of sex [*F*_(1, 75)_ = 662.6, *p* < 0.0001] as well as interaction between sex and diet [*F*_(1, 75)_ = 12.51, *p* = 0.0007].

Additionally, while both sexes of HFD-fed rats show an increased energy intake per gram body weight, the female calorie consumption is slightly, but significantly, higher when the daily energy intake after both 2 and 10 weeks on the diet is normalized to the body weight [for 2 weeks: effect of diet *F*_(1, 76)_ = 48.94, *p* < 0.0001, effect of sex *F*_(1, 76)_ = 14.09, *p* = 0.0003; for 10 weeks: effect of diet *F*_(1, 75)_ = 25.97, *p* < 0.0001, effect of sex *F*_(1, 75)_ = 8.901, *p* = 0.0038; Figure 1H]. As the rats were pair-housed, the calculations were made based on the average food intake of each cage, normalized to the individual body weights.

Likewise, white adipose tissue depots, when measured as absolute weight, were not heavier in females fed an obesogenic diet compared to their chow controls (Figures 1I,J). Male rats fed a high-fat diet, however, showed a 2-fold increase of both gonadal and inguinal fat mass compared to chow-fed controls [two-factor ANOVA for IWAT: interaction *F*_(1, 74)_ = 23.17, *p* < 0.0001, effect of diet *F*_(1, 74)_ = 44.50, *p* < 0.0001, effect of sex *F*_(1, 74)_ = 118.2, *p* < 0.0001; for GWAT: interaction *F*_(1, 74)_ = 18.59, *p* < 0.0001, effect of diet *F*_(1, 74)_ = 43.98, *p* < 0.0001, effect of sex *F*_(1, 74)_ = 118.1, *p* < 0.0001; Figures 1I,J].

When normalizing the weight of the fat depots to the final body weight of the animals, the data reveal a significant interaction between diet and sex for both adipose fat depots. Even when expressed as % of body weight, males gained substantially more IWAT and GWAT compared to female rats. Both fat pads were still nearly twice as heavy in males fed the obesogenic diet, vs. a 30% trend to increase in IWAT and a 60% increase in GWAT in females [IWAT: interaction *F*_(1, 74)_ = 8.453, *p* = 0.0048, effect of diet *F*_(1, 74)_ = 31.66, *p* < 0.0001, effect of sex *F*_(1, 74)_ = 30.05, *p* < 0.0001; for GWAT: interaction *F*_(1, 74)_ = 4.007, *p* = 0.049, effect of diet *F*_(1, 74)_ = 34.06, *p* < 0.0001, effect of sex *F*_(1, 74)_ = 24.03, *p* < 0.0001; Figures 1K,L]. At baseline (i.e., on chow diet) there was no significant difference in the % of IWAT between males and females, males, however had more GWAT.

Despite the large increase in fat mass and food intake in males, glucose tolerance remained unaffected when challenged with an oral administration of glucose after 12 weeks on the diet (Figure 1M). Neither sex displayed a change in peak glucose levels or glucose clearance, however blood glucose levels were consistently higher in males than females throughout the test (Figure 1N).

### Energy Expenditure Differs Between Male and Female Rats: Activation of Brown Adipose Tissue Thermogenesis in Response to a High-Fat Choice Diet Challenge Is Dictated by Sex

**Figure 2:**
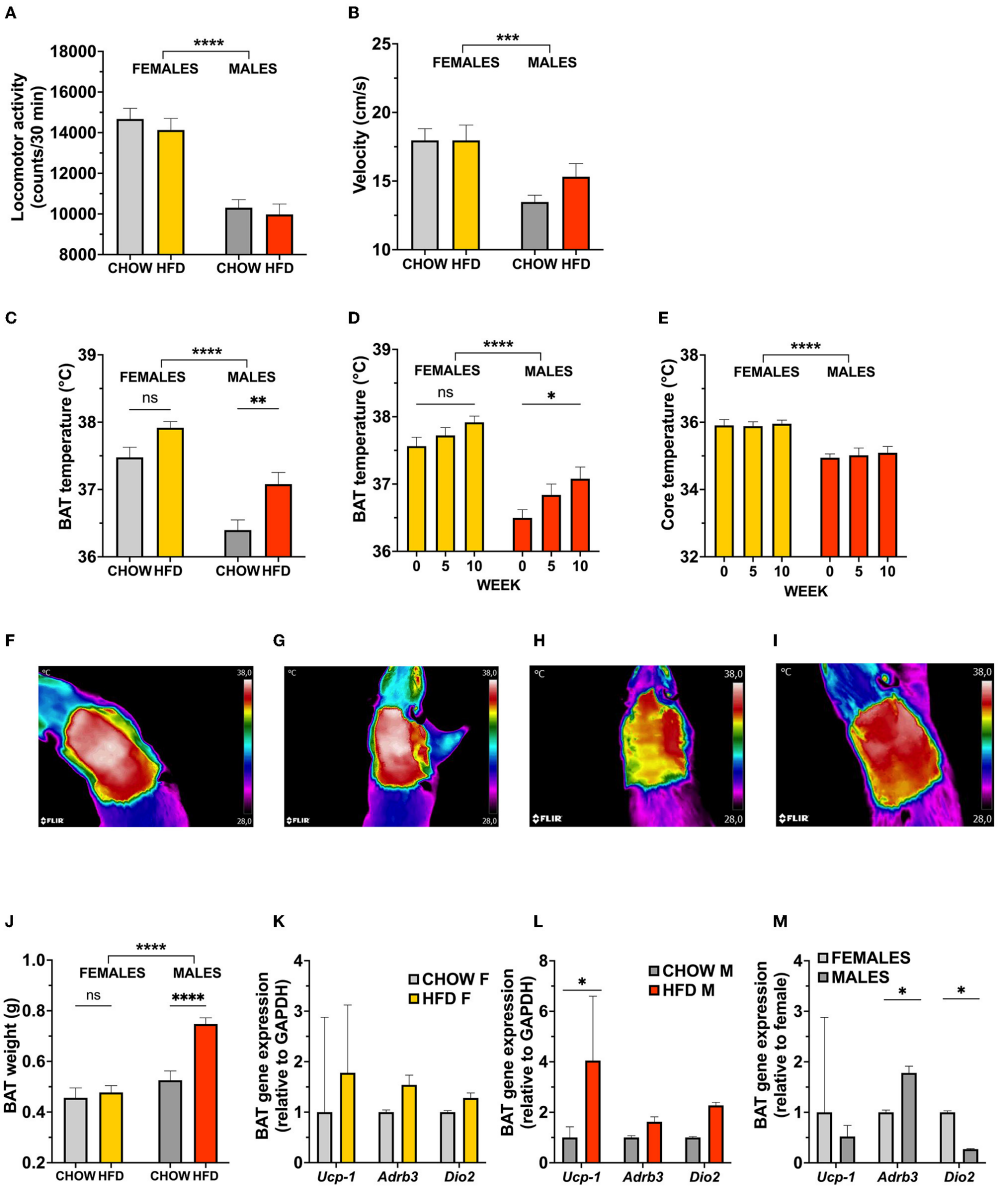
Activation of brown adipose tissue thermogenesis in response to a high-fat choice diet challenge is dictated by sex in rats. Male locomotor activity **(A)**, and velocity **(B)** was significantly higher in female rats, but remained unaffected by diet in either sex when measured during a 30-min test. Furthermore, infrared images reveal a higher basal core temperature and thermogenic activity of BAT in females, which remains statistically unaltered with HFD **(C)**. In contrast, high-fat fed male rats showed a potent increase in BAT temperature **(D)**, and BAT weight **(J)**, with no subsequent changes in core temperature **(E)**. Representative infrared images of BAT region in female (**F,G**; chow and HFD, respectively) and male (**H,I**; chow and HFD, respectively) rats. Increased BAT temperature activity in males (*n* = 8 per diet group) coincided with elevated BAT thermogenesis related gene expression levels **(L)**. No changes were detected in female (*n* = 9 per diet group) gene expression levels **(K)**. Expression of BAT genes differ at baseline in female and male rats **(M)**. Data are expressed as mean ± SEM. *p < 0.05, **p < 0.01, ***p < 0.001, ****p < 0.0001 compared with respective controls. *n* = 40 (females, 20 in each diet group); *n* = 39 (males, 19 in HFD group). HFD, high-fat diet; BAT, brown adipose tissue; GAPDH, glyceraldehyde-3-phosphate dehydrogenase (housekeeping gene); *Ucp1*, uncoupling protein 1; *Adrb3*, Beta-3 adrenoreceptor; *Dio2*, Iodothyronine Deiodinase 2.

Energy intake excess has been demonstrated to stimulate adaptive responses in energy expenditure (26). This prompted us to assess whether locomotor activity and BAT activity were altered in rats fed an obesogenic diet. After 11 weeks on the diet, locomotor activity and velocity of HFD-fed males and females remained unaltered (Figures 2A,B). It is interesting to note that, similarly to the higher basal thermogenesis presented in females, there is an effect of sex at baseline in both amount of movement and speed of locomotion [two-factor ANOVA for locomotor activity: effect of sex *F*_(1, 74)_ = 70.87, *p* < 0.0001; for velocity: effect of sex *F*_(1, 74)_ = 16.24, *p* < 0.0001; Figures 2A,B]. We additionally analyzed the data from the locomotor test for anxiety-like behavior, to control for confounding effects of the novel environment. The arena was virtually divided into center and periphery, to score time spent in each compartment. There were no diet-associated changes in anxiety-like behavior in either sex, time spent in center was 52.42 and 56.43 s for females (chow vs. HFD, respectively; SE = 7.094), and 32.43 and 31.61 s for males (chow vs. HFD, respectively; SE = 7.288) [two-factor ANOVA for time spent in center: effect of diet *F*_(1, 70)_ = 0.0982, *p* = 0.7548, effect of sex *F*_(1, 70)_ = 19.41, *p* < 0.0001].

Utilizing infrared imaging (FLIR^TM^), BAT and core temperature were measured prior to, as well as 5 and 10 weeks after, exposure to HFD. Thermal imaging revealed that HFD increased BAT temperature only in males (Figures 2C,D, representative images: Figures 2H,I) reaching significance after 10 weeks on the diet. Two-way repeated measures ANOVA indicated that the main effect of sex on BAT temperature was significant [*F*_(1, 75)_ = 43.86, *p* < 0.0001], where female rats had overall higher BAT temperature prior to the diet exposure, a temperature which remained high during HFD. We also found an effect of diet [*F*_(1, 75)_ = 15.02, *p* = 0.0002]. There was no significant interaction between these two factors [*F*_(1, 75)_ = 0.7002, *p* = 0.4054]. This increase was not associated with changes in core temperature (Figure 2E), indicating that the thermogenic adaption is BAT-specific and likely does not change the thermoregulatory set point, which would be reflected in changes in core temperature. Even though female BAT temperature tended to be increased in response to HFD, this difference did not reach significance (*p* = 0.0663; Figure 2C, representative images: Figures 2F,G). Considering the chronically higher BAT temperature displayed by females, a ceiling effect in the thermogenic potential is a possible explanation for a lack of diet effect.

In line with the sex-specific effect of diet induced thermogenesis, a large increase in BAT mass is seen in male, but not female, rats [two-factor ANOVA: interaction *F*_(1, 74)_ = 0.236, *p* = 0.0033, effect of diet *F*_(1, 74)_ = 13.63, *p* = 0.0004, effect of sex *F*_(1, 74)_ = 26.66, *p* < 0.0001; Figure 2J]. With the purpose of unraveling whether this growth is related to an increase in thermogenic potential, expression of genes involved in thermogenic control was measured. Consistent with the elevations in BAT temperature, high-fat diet significantly increased the expression of *Ucp1* (uncoupling protein 1, responsible for thermogenic respiration) in males (*p* = 0.01, Figure 2L). In female rats, no changes were found in the expression of *Ucp1, Dio2* (iodothyronine deiodinase 2, responsible for local production of bioactive thyroid hormone) or *Adrb3r* (β_3_-adrenergic receptor; stimulates BAT thermogenesis upon activation) (Figure 2K). However, in comparison to males, chow-fed females show a higher expression of Dio2, as well as a trend in higher *Ucp1* expression (*p* = 0.001 and *p* = 0.08, respectively, Figure 2M).

### Male and Female Mice Show Greater Consequences for Metabolic Disease Progression in Response to a High-Fat Choice Diet Challenge

**Figure 3:**
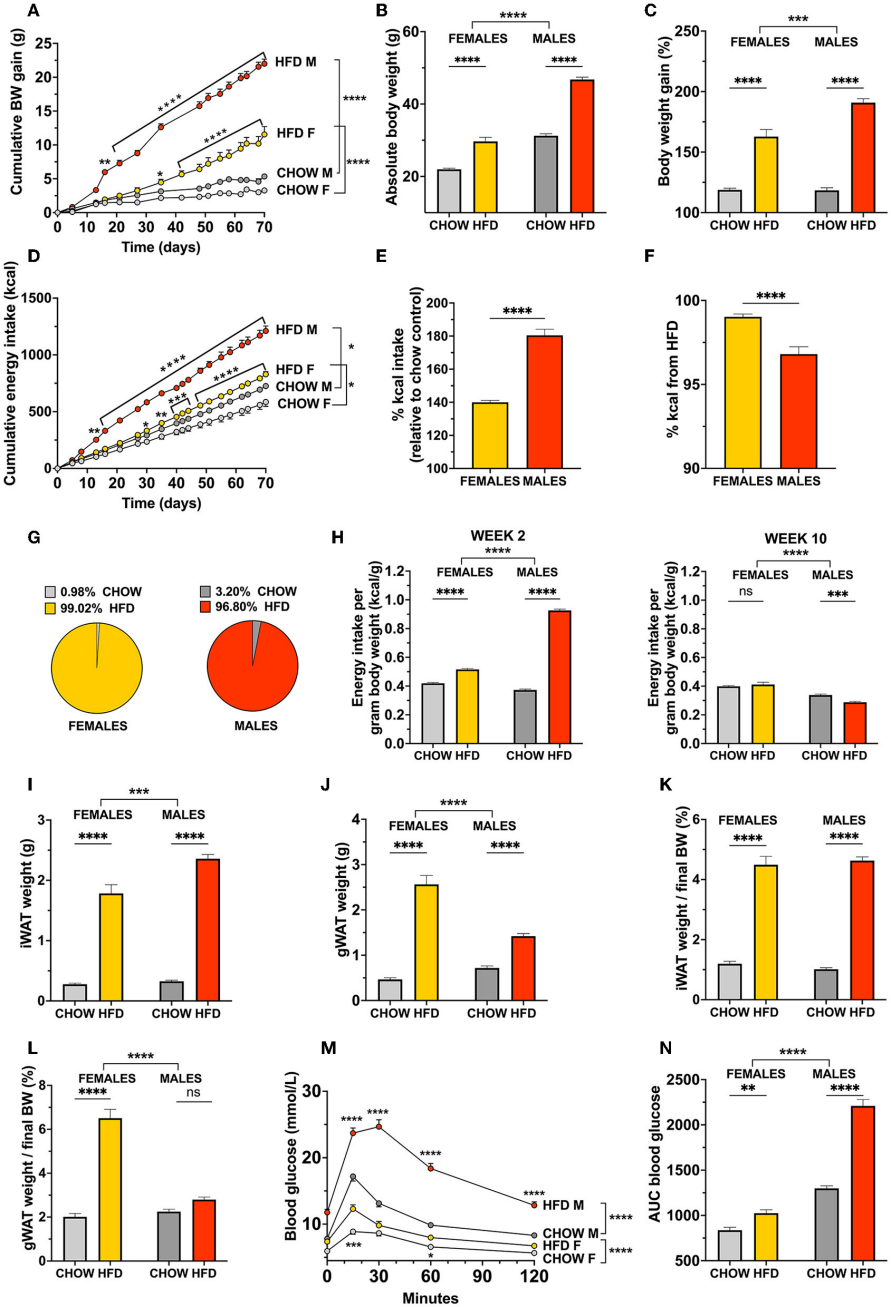
Sex divergent effect of a high-fat choice diet challenge in mice. When offered a high-fat and chow choice diet, mice responded with a pronounced hyperphagia **(D)** and weight gain **(A)** that had a more rapid onset in males compared to females. In contrast to rats, both female and male mice presented a dramatic increase in body weight gain **(B,C)**, overeating **(E)**, adiposity **(I–L)**. Female mice gained 3-fold more visceral fat compared to males **(L)**. Both sexes showed impaired glucoregulation, although even here blood glucose levels and clearance a determined by AUC were significantly more affected by the obesogenic diet in males **(M,N)**. Similar to female rats, female mice showed a higher preference for HFD than males **(F)**, but in comparison to rats the proportion of chow consumed is drastically smaller in both sexes **(G)**. After 2 weeks on the diet, both female and male mice consumed more energy per gram body weight than their chow counterparts. However, at the final week of the study female mice consumed more energy per gram of body weight than male mice irrespective of the diet and no significant effect of diet was found (**H**; left and right, respectively). Data are expressed as mean ± SEM. *p < 0.05, **p < 0.01, ***p < 0.001, ****p < 0.0001 compared with respective controls, in **(A,D,M)** the pairwise comparisons indicate the comparison between diet groups within each sex *n* = 40 (females, 20 in each diet group); *n* = 40 (males, 20 in each diet group). BW, body weight; HFD, high-fat diet; IWAT, inguinal (subcutaneous) white adipose tissue; GWAT, gonadal white adipose tissue; AUC, area under the curve.

Next, we investigated if the sex differences in diet-induced obesity observed in rats, can also be identified in mice offered the same high-fat diet along with normal chow. Ingestive behavior followed a similar pattern as that obtained from the rat group, and male mice gained weight more rapidly on the HFD (Figures 3A,D). In fact, female mice did not reach a significant increase in cumulative body weight gain until more than 3 weeks later (day 42, *p* = 0.001; Figure 3A). Though marked and significant in both sexes, male mice showed a higher level of overeating than females (*p* < 0.0001; Figure 3E), when comparing the average energy intake per cage over the 10-week period. This hyperphagia consequently resulted in a body weight that was almost doubled in males, and clearly elevated in females, both when assessed as absolute body weight [two-factor ANOVA: interaction *F*_(1, 76)_ = 29.56, *p* < 0.0001, effect of diet *F*_(1, 76)_ = 259.5, *p* < 0.0001, effect of sex *F*_(1, 76)_ = 334.1, *p* < 0.0001; Figure 3B], and as the percent of starting body weight [two-factor ANOVA: interaction *F*_(1, 76)_ = 15.54, *p* = 0.0002, effect of diet *F*_(1, 76)_ = 260.2, *p* < 0.0001, effect of sex *F*_(1, 76)_ = 14.70, *p* = 0.0003; Figure 3C]

In line with the results seen in rats, female mice, compared to males, had a higher preference to consume the palatable food over the chow, but this difference was not as pronounced in mice (*p* < 0.0001, Figure 3F). In stark contrast to rats, mice offered a choice diet almost exclusively elected to ingest the HFD, with both sexes consuming more than 95% of their daily intake from the palatable food (Figure 3G).

At the initial stages of overeating, both sexes consumed more calories per gram body weight compared to their chow counterparts, although this was remarkably more pronounced in males [for 2 weeks: interaction *F*_(1, 76)_ = 1122, *p* < 0,0001, diet *F*_(1, 76)_ = 2269, *p* < 0,0001, sex *F*_(1, 76)_ = 709.4, *p* < 0,0001; Figure 3H, left]. After 10 weeks on the diet, female mice consumed more calories per gram body weight, similarly to the rats, and a two-factor ANOVA analysis revealed only a trend in effect of diet, but a significant effect of sex, and a significant interaction [interaction *F*_(1, 76)_ = 10.78, *p* = 0.0016, diet *F*_(1, 76)_ = 3.781, *p* = 0.0556, sex *F*_(1, 76)_ = 92.18, *p* < 0.0001; Figure 3H, right]. Surprisingly, only the obese male mice showed a reduced energy intake per gram body weight, indicating a compensation in consummatory behavior over time. It should be noted that the mice were housed in groups of 10 which disabled individual food intake measures, therefore, we calculated the average intake per mouse in each cage, and normalized it to the individual body weights.

The excessive body weight gain in male and female mice was reflected by a robustly increased adiposity, with more than a 7-fold increase in mass of subcutaneous inguinal white adipose tissue (IWAT) compared to their respective chow-fed controls [two-factor ANOVA: interaction *F*_(1, 76)_ = 10.42, *p* = 0.0018, effect of diet *F*_(1, 76)_ = 468.2, *p* < 0.0001, effect of sex *F*_(1, 76)_ = 14.61, *p* = 0.0003; Figure 3I]. The results did not differ when IWAT weight was calculated as gram per gram pf body weight, i.e., it was equally increased in both males and females [IWAT: effect of diet *F*_(1, 76)_ = 462.6, *p* < 0.0001]. Furthermore, when analyzed as absolute weight, visceral fat was also significantly expanded in both sexes. This change was much more dramatic in HFD females, which had a 5-fold heavier gonadal adipose white tissue (GWAT) than chow-fed controls, and ultimately a higher GWAT weight than obese males [two-factor ANOVA: interaction *F*_(1, 76)_ = 43.09, *p* < 0.0001, effect of diet *F*_(1, 76)_ = 173.3, *p* < 0.0001, effect of sex *F*_(1, 76)_ = 17.41, *p* < 0.0001; Figure 3J]. For GWAT expressed as % of body weight revealed no effect of diet in male but still a dramatic fat gain in females [interaction *F*_(1, 76)_ = 73.9, *p* < 0.0001, effect of diet *F*_(1, 76)_ = 121.0, *p* < 0.0001, effect of sex *F*_(1, 76)_ = 57.35, *p* < 0.0001; Figures 3K,L]. Interestingly, and in contrast to rats, the percent body fat in chow-fed mice was similar between sexes in both IWAT and GWAT.

Impaired glucose tolerance was identified in both sexes. Fasting blood glucose levels were elevated in male and female mice fed HFD (*p* = 0.0362 and *p* < 0.0001, for females and males, respectively; Figure 3M). Correspondingly, peak glucose levels during an OGTT were markedly higher compared to controls, males and females alike (*p* < 0.0001 and *p* < 0.0001, for females and males, respectively). Males had a greater delay in glucose clearance, and their glucose levels did not return to baseline levels at the 120 min time point (Figure 3M). Accordingly, the increase in AUC value was more pronounced in HFD males than females. Consistent with rat data, female mice displayed lower fasting blood glucose and AUC value than males, regardless of diet [two-factor ANOVA: interaction *F*_(1, 76)_ = 65.10, *p* < 0.0001, effect of diet *F*_(1, 76)_ = 149.8, *p* < 0.0001, effect of sex *F*_(1, 76)_ = 337.2, *p* = 0.0003; Figure 3N]. The effect of the obesogenic diet on AUC for blood glucose was 2-fold larger in males compared to females (Figure 3N).

### Energy Expenditure Is Reduced in Obese Male and Female Mice

**Figure 4:**
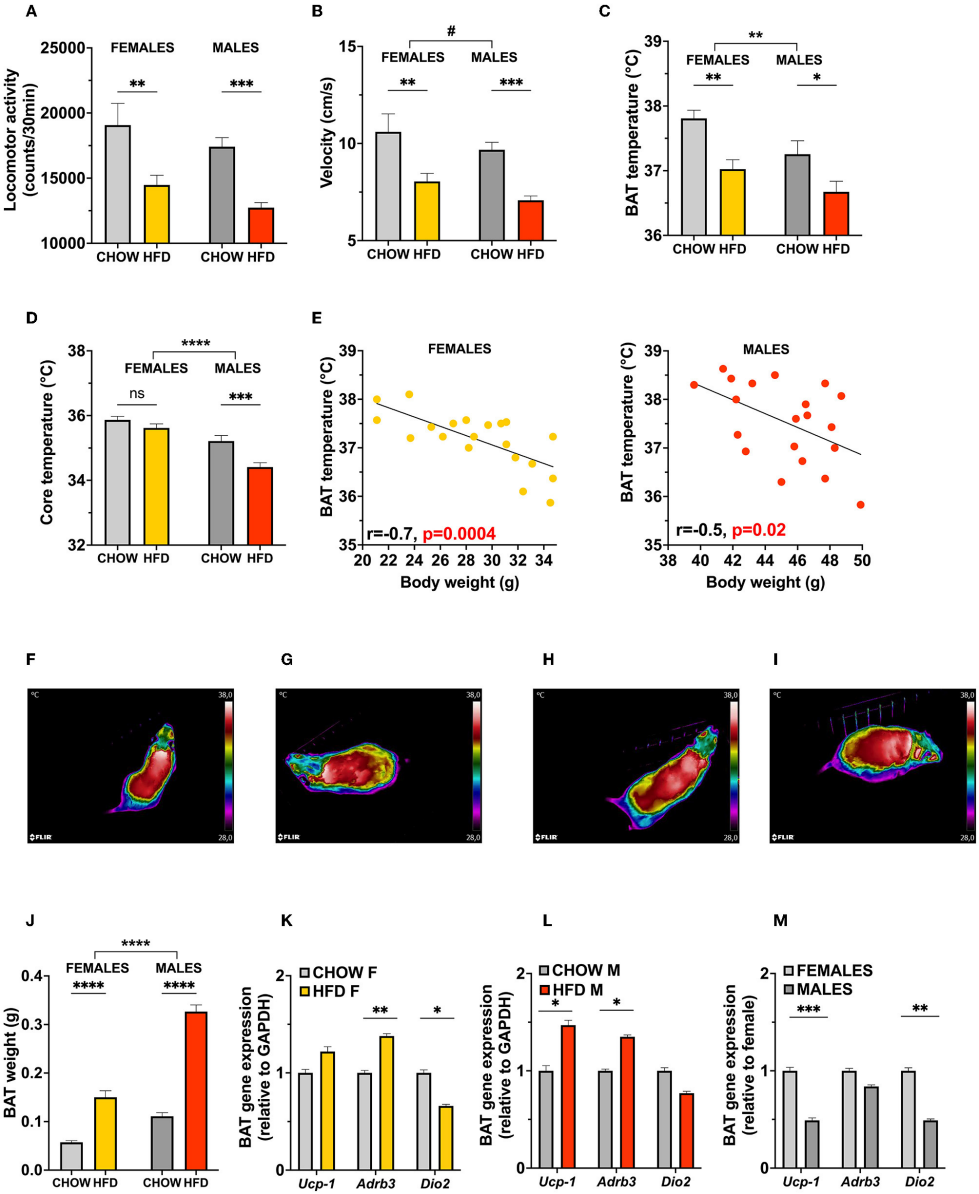
Energy expenditure is reduced in obese male and female mice. Increased body weight of HFD mice lead to a reduction in locomotor activity **(A)** and velocity **(B)** in both males and females, with no significant differences found at baseline between the two sexes. In line with the sex-specific thermoregulation shown in rats, female mice displayed a higher basal BAT and core temperature **(C,D)**. However, an opposite effect of diet is observed in mice, where high-fat diet reduced BAT region temperature in both sexes **(C)**. Linear regressions of BAT temperature with body weight as a covariate in female and male mice on HFD (**E**; left and right, respectively) reveals an inverse relationship between weight gain and temperature of BAT region. Representative infrared images of BAT region in female (**F,G**; chow and HFD, respectively) and male (**H,I**; chow and HFD, respectively) mice. HFD increases BAT weight in both sexes **(J)**, and affects BAT thermogenesis related gene expression levels in female **(K)** and male **(L)** mice. Expression of BAT genes differ at baseline in female and male mice **(M)**. Data are expressed as mean ± SEM. *p < 0.05, **p < 0.01, ***p < 0.001, ****p < 0.0001 compared with respective controls. *n* = 40 (females, 20 in each diet group); *n* = 40 (males, 20 in each diet group). HFD, high-fat diet; BAT, brown adipose tissue; GAPDH, glyceraldehyde-3-phosphate dehydrogenase (housekeeping gene); *Ucp1*, uncoupling protein 1; *Adrb3*, Beta-3 adrenoreceptor; *Dio2*, Iodothyronine Deiodinase 2.

In contrast to rats, locomotor activity and velocity were altered by the obesogenic diet in mice (Figures 4A,B). No sex differences were observed at baseline, but both male and female HFD-fed mice showed a clear decrease in spontaneous activity [two-factor ANOVA for locomotor activity: effect of diet *F*_(1, 74)_ = 23.77, *p* < 0.0001, no significant effect of sex or interaction between the two factors; for velocity: effect of diet *F*_(1, 74)_ = 23.78, *p* < 0.0001, no significant effect of sex or interaction between the two factors; Figures 4A,B]. There were no diet-associated changes in anxiety-like behavior in either sex, time spent in center was 144.7 and 152.1 s for females (chow vs. HFD, respectively; SE = 22.02), and 156.4 and 139.4 s for males (chow vs. HFD, respectively; SE = 21.43) [two-factor ANOVA for time spent in center: effect of diet *F*_(1, 74)_ = 0.09628, *p* = 0.7572, effect of sex *F*_(1, 74)_ = 0.001035, *p* < 0.9744].

Surprisingly, BAT temperature was decreased in both males and females after 10 weeks of exposure to HFD diet [two-factor ANOVA: effect of diet *F*_(1, 76)_ = 17.22, *p* < 0.0001, effect of sex *F*_(1, 76)_ = 7.568, *p* = 0.0074, no significant interaction between the two factors; Figure 4C, representative images: Figures 4F–I]. This change was more pronounced in females, however, only in males was it associated with a decrease in measured core temperature [two-factor ANOVA: interaction *F*_(1, 76)_ = 4.084, *p* = 0.0468, effect of diet *F*_(1, 76)_ = 14.58, *p* = 0.003, effect of sex *F*_(1, 76)_ = 45.60, *p* < 0.0001; Figure 4D]. Linear regressions of BAT temperature with body weight as a covariate in female and male mice on HFD (Figure 4E; left and right, respectively) revealed an inverse relationship between body weight and temperature of BAT region (*p* = 0.0004, r = −0.7 and *p* = 0.02, r = −0.5); for males and females, respectively.

Gene expression analysis indicated an elevation of *Ucp1* and *Adrb3* expression in male mice (*p* = 0.014 and *p* = 0.009, respectively, Figure 4L), while females presented with only an elevation of *Adrb3* and a significant reduction of *Dio2* expression (*p* = 0.028 and *p* = 0.05, respectively, Figure 4K). However, in comparison to females, chow-fed male mice showed lower expression of *Ucp1* and *Dio2* (*p* < 0.0001 and *p* = 0.04, respectively, Figure 4M).

## Discussion

Animal models of diet-induced obesity are a crucial tool for investigating energy balance failure in the presence of highly palatable food. How the response to an obesogenic diet differs between sexes and rodent species maintained under the same conditions is still, surprisingly, poorly understood. Therefore, in the current study we evaluated impact of sex, and species, on energy balance in response to a chronic energy-dense diet. The findings reveal stunning sex divergence and species divergence in feeding behavior and energy expenditure responses to diet-induced obesity challenge.

Rats and mice were given a free choice of HFD and chow, a model that better mimics human exposure to a variety of food choices than the more commonly used exclusive HFD feeding strategy (27). As expected, an increase in caloric intake was observed in all animals offered HFD. However, the expression of hyperphagia and weight gain was sexually dimorphic, in rats and mice alike. Males displayed greater caloric intake and body weight gain than females when offered HFD, irrespective of species.

Rather few studies evaluating DIO include female subjects and are powered to analyze effect of sex, and the few that have, show conflicting outcomes. Overall, in studies where rats are offered sweet solutions (sucrose water or sweet milk), females show a greater hyperphagic response (10, 28, 29), while males only present with a persistent overconsumption and fat gain on a HFHS (high-fat high-sugar) paradigm (27, 30). Moreover, Taraschenko et al. demonstrated how the choice of palatable diet produces a contrasting sex divergent response; while a HFD (40%) induced weight gain in males only, the opposite was true for a high sucrose diet (11, 31). Therefore, combination of fat and sugar seems to be important for the induction of hyperphagia, especially in male rats.

Although female rats and mice in our study displayed a lower level of overeating than males, they did show a higher preference for the HFD. This suggests that female rats regulate their energy intake better than males, due to a better ability to compensate for the higher energy density of the diet. Higher preference for the palatable diet in females may indicate a more hedonically-driven feeding behavior in this sex. This is supported by a previous study, where female rats show a stronger shift in preference palatable food in a conditioned place preference paradigm and a higher activation of brain regions linked to hedonic, but not homeostatic, regulation of food intake in females, compared to males (32).

While mice also displayed a strong preference for HFD, the extent of their preference was astonishing; up to 99% of total intake came from the palatable diet. One of the few other studies offering a free choice HFD to mice, showed that chow is kept as a major portion of daily intake when mice are offered lard and sucrose (33). Commercial high-fat diet, as the one used in the current study, has a protein content that meets nutritional needs, implying the importance of the macronutrient profile in palatability-driven overconsumption. Interestingly, mice showed an inferior ability to compensate for the higher energy density of the palatable diet, and ingested a pellet mass comparable to controls.

Although male mice gained more weight, and almost tripled their white adipose tissue depots, it was the females that obtained a remarkably abnormal deposition of adiposity. Visceral obesity is associated with metabolic disturbances, and female mice in the current study displayed a striking 5-fold increase in a visceral, gonadal, adipose tissue compared to “only” a 2-fold increase seen in male mice. It is possible that the surprisingly modest GWAT mass detected in males may be a result of increased adipocyte death (34), and that the weight is poorly reflecting adipocyte dysfunction. GWAT accumulation has been shown to play an important role in glucose homeostasis, especially in females, as partial removal improves glucose tolerance in both lean and HFD-fed female mice (35). Our data, in addition to recent studies, suggest that the less severe metabolic responses often reported in female mice can be explained by the duration of diet exposure and the age at diet intervention (14, 15).

Collectively, we show that mice responded to the metabolic challenge with a more severe metabolic phenotype leading both sexes to accumulate visceral fat and impaired glucose metabolism, but with females showing a much higher accumulation of visceral fat. In stark contrast, female rats appeared protected from an obese phenotype, which could be partially explained by the less pronounced hyperphagia and the tendency to consume more calories from chow, compared to female mice.

Increasing physical activity represents a potential compensatory mechanism to expand the extra calories consumed, therefore we measured locomotion in mice and rats. We found robust sex differences irrespective of the diet, where female rats moved nearly 50% more than males, and moved much faster than males as well. This is consistent with previous research showing that females are more active than male rats (10), and may partially explain the less pronounced weight gain seen in female rats. Surprisingly, locomotor activity was not affected by the diet in either sex. In contrast, mice did not display any sex difference in locomotion, and both sexes moved less and slower when maintained on the HFD, suggesting that in mice not only is activity failing to compensate for the excessive caloric intake, but it may contribute to weight gain by further tipping the scale toward positive energy balance. There is previous evidence that female mice temporarily increase locomotor activity after 5 weeks on HFD, but this compensatory activity is diminished after a more chronic exposure (16, 36, 37). This diet-induced hypoactivity in mice may be a result of the physical restrictions caused by the severe fat accumulation. However, it is important to note that spontaneous physical activity should be measured over a longer duration of time and preferably in the home cage of the animal. Thus, the activity measured here is more representative of novelty-induced locomotor activity rather than spontaneous activity. Although we did not find any diet-associated changes in anxiety-like behavior in either sex or species, it is conceivable that spontaneous activity measured in the familiar environment of the home cage and taken over a longer period of time would reveal different results.

Another possible explanation for the obesity resistance we observed in female rats, is higher energy expenditure due to BAT thermogenesis. Here, we show that female rats have a significantly higher BAT temperature than male rats. While male rats on average had 50% of *Ucp1* and 20% of *Dio2* expression compared to females, this was not significant, likely due to large variability in expression of both these genes in males. Others have shown that females have greater thermogenic capacity, and higher gene expression and protein levels of Ucp1 in BAT (38–41).

We found that only male rats increased BAT thermogenesis progressively in response to HFD challenge. Overfeeding is a state of high adrenergic stimulation, and the higher levels of β_3_-adrenergic receptor expression in chow-fed males could be contributing to the male specific diet-induced thermogenesis. The sexually dimorphic activation of BAT in response to an obesogenic diet is line with previous observations (12, 38). In contrast to current data, these studies report a lesser weight gain in males than females. This inconsistency may be attributed the difference in rat strain (Wistar vs. SD), diet (cafeteria diet vs. HFD), or age of diet onset (10 and 30 days, respectively, vs. 10 weeks old). Additionally, while BAT temperature increased in male rats in response to the HFD, core temperature did not, suggesting that the thermoregulatory set point is not altered by the diet, but rather a selective increase in BAT activity is compensated for by heat dissipation elsewhere, likely by vasodilation (42).

These data indicate that there may be a sex divergent role for BAT thermogenesis in the regulation of whole-body energy homeostasis, where female rats were less susceptible to an excessive weight gain due to an inherently higher BAT activity, whereas males were more inclined to engage in a compensatory, but insufficient, energy expenditure strategy in response to overconsumption. It is also possible that a certain level of hyperphagia has to be reached to engage diet-induced thermogenesis, a level that may not have been reached by female rats. Moreover, females may have a lower threshold temperature for BAT activation, as room temperature (21–22°C) is below rodent thermoneutrality (43). While the BAT activation in males was not sufficient to prevent excessive weight gain and adiposity, it might have contributed to maintaining a normal level of glucose tolerance. This hypothesis is supported by a wealth of literature suggesting that BAT activation can normalize glucoregulation in obesity (44–46).

In contrast to results obtained in rats, and also in contrast to the idea that BAT is engaged as a compensatory mechanism during excessive feeding, HFD challenged mice had a pronounced decrease in BAT temperature. Furthermore, there was a tight correlation between BAT temperature and body weight: the higher the reduction in BAT thermogenesis the higher body weight in both male and female mice. It is plausible that the severity of obesity seen in mice has resulted in BAT hypotrophy, BAT “whitening” (or lipid deposition) and loss of function. Another plausible explanation is that an increase in subcutaneous fat covering the thermogenic tissue could potentially mask the true, possibly increased, temperature. However, BAT gene expression analysis suggests a more nuanced response, *Ucp1* gene expression was increased only in HFD-fed males, while β_3_-adrenergic receptor expression was increased in both sexes. The increased expression of β_3_-adrenergic receptor in male mice could point to an increased sympathetic input, given also the increased *Ucp1* in males. Thus, it is possible that in males BAT has increased thermogenic capacity that we were unable to detect with the thermal imaging. Yet in females, given the lack of *Ucp1* changes, the increased receptor expression could indicate compensatory feedback on receptor level due to reduced noradrenaline stimulation. This is also in line with a reduced thyroid input to BAT in females, but not males, as indicated by reduced *Dio2* expression. An abnormal thyroid activation of BAT contributes to lower sensitivity to noradrenaline stimulation and lower thermogenic capacity (47). Despite lower BAT temperature, most likely a result of inadequate synthesis of active thyroid hormone, and lower locomotor activity during HFD challenge, female mice preserved their core temperature at the level of chow-fed controls. Several mechanisms could allow for this, mostly simply the increased subcutaneous adipose tissue likely provided ample insulation. It is also possible that female mice were able to increase WAT beige-ing (48, 49), alternatively that they conserved heat *via* vasocontraction (50) to preserve the thermoregulatory set point.

Many of the established differences in obesity development are attributed to sex steroid hormones (2), specifically the protective role of estrogens (17). Male rats are more sensitive to the catabolic effects of insulin, whereas females are more sensitive to that of anorexic leptin, an effect determined by the central effects of estrogens (51). Generally, estradiol acts to suppress feeding by enhancing the potency of anorexic food control signals (52–54), and by decreasing the potency of orexigenic signals (55, 56). Additionally, estradiol has been identified as a key modulator of BAT activity, and central administration of estradiol in female rats, leads to increased BAT *Ucp1* mRNA expression and core body temperature (57). Thus, it is likely that the differential food selection patterns between the sexes, different degree of overeating, as well as differential thermogenic response to DIO are at least partly driven by gonadal hormones. Although, interestingly, mouse and rat females are likely to have similar gonadal steroid fluctuations, and presumably largely similar sex steroid receptor distribution, yet disparate responses to the obesogenic diet.

In summary, we show that diet-induced hyperphagia is greater in males offered a free-choice of HFD and chow, irrespective of species, and that females display a higher preference for the palatable food. Our results indicate that female rats show a delay in diet-induced obesity and metabolic complications due to a higher energy expenditure and lower level of hyperphagia. Although male rats engage in a compensatory energy expenditure strategy, this fails to rescue them from adiposity, but might contribute to maintaining a healthy glucose tolerance despite overeating. Furthermore, female and male mice presented with a dramatic adiposity and impaired glucose tolerance, possibly due to a decreased energy expenditure and to greater intake of saturated fats that are linked to escalating metabolic consequences.

Prevalence of overweight and obesity differs by sex. Girls are less likely to be obese compared to boys (ages 5–19), women are less likely to be overweight than men, and premenopausal women tend to have lower rates of obesity (58). Yet, women are the fastest growing constituent of obese individuals and have almost double the risk to develop extreme obesity, according to CDC. Thus, it appears that women may be less susceptible to the onset diet-induced weight gain, especially prior to menopause, but once it emerges there is an escalating response. It is noteworthy that non-biological factors, such as socioeconomic status, may be driving some of the human sex differences. Factors such as income and education are still distributed differently between genders in most countries, contributes to human obesity, and are challenging to include in animal models. We report remarkable sex differences in response to an obesogenic environment, signifying the importance of studying both males and females to understand differences in energy homeostasis, and potentially develop more effective, sex tailored, weight loss strategies. Our data suggest that rats on a free choice HFD, where females were less likely to gain weight and gained less adiposity, show better face validity for the protective effect of female sex in girls and younger women, and for the lower prevalence of overweight in women overall. Female mice did display a remarkable accumulation of visceral fat that surpassed the male fat mass, showing a better face validity for modeling more severe obesity in women. Thus, depending on the aspect and stage of human obesity that is to be studied, rats or mice may be a more fitting animal model.

## Data Availability Statement

The original contributions presented in the study are included in the article/Supplementary Materials, further inquiries can be directed to the corresponding author/s.

## Ethics Statement

The animal study was reviewed and approved by Animal Welfare Committee of the University of Gothenburg, Sweden, Ethical permit # 137/15.

## Author Contributions

J-PK, KS, IW, and IM contributed to conception and design of the study. J-PK, PV, and IM carried out the experiments. J-PK, PV, SB, MA, MV, and IM contributed to sample collection. J-PK, KS, PV, and IM processed the experimental data, performed the analysis, drafted the manuscript, and designed the figures. All authors contributed to manuscript revision, read, and approved the submitted version.

## Funding

This research was funded by the Wallenberg Foundation (WCMTM), Swedish Research Council (2018-00660 to KS), Ragnar Söderberg Foundation (KS), and the Swiss National Science Foundation (183899 to J-PK). IW was funded by Swedish Research Council (2013-07107, 2017-00792, and 2020-01463), the NovoNordisk Foundation (NNF19OC0056601), and the Swedish Diabetes Foundation (DIA2019-419).

## Conflict of Interest

The authors declare that the research was conducted in the absence of any commercial or financial relationships that could be construed as a potential conflict of interest.

## Publisher's Note

All claims expressed in this article are solely those of the authors and do not necessarily represent those of their affiliated organizations, or those of the publisher, the editors and the reviewers. Any product that may be evaluated in this article, or claim that may be made by its manufacturer, is not guaranteed or endorsed by the publisher.
