# Supplementary material for: Sex and Species Differences in the Development of Diet-Induced Obesity and Metabolic Disturbances in Rodents
Source: Front Nutr. 2022 Feb 17;9:828522. doi: 10.3389/fnut.2022.828522 (PMC8908889; doi:10.3389/fnut.2022.828522)
Supplement: Supplementary file 1 [file Data_Sheet_1.PDF]

Location of graphed data	Parameters measured	3-way ANOVA results		
Fig 1C, 3C	Body weight gain (%)	Species x diet x sex	F (1, 151) = 2,707	P=0,1020
		Species x diet	F (1, 151) = 76,33	P<0,0001
		Species x sex	F (1, 151) = 21,34	P<0,0001
		Diet	F (1, 151) = 283,1	P<0,0001
		Sex	F (1, 151) = 114,4	P<0,0001
		Species	F (1, 151) = 14,64	P=0,0002
Fig 1E, 3E	% kcal intake	Species x diet x sex	F (1, 89) = 14,86	P=0,0002
		Species x diet	F (1, 89) = 96,97	P<0,0001
		Species x sex	F (1, 89) = 14,86	P=0,0002
		Diet	F (1, 89) = 451,8	P<0,0001
		Sex	F (1, 89) = 43,57	P<0,0001
		Species	F (1, 89) = 96,97	P<0,0001
Fig 1F, 3F	% kcal from HFD	Species x diet x sex	F (1, 104) = 36,56	P<0,0001
		Species x diet	F (1, 104) = 4022	P<0,0001
		Species x sex	F (1, 104) = 36,56	P<0,0001
		Diet	F (1, 104) = 5317	P<0,0001
		Sex	F (1, 104) = 123,4	P<0,0001
		Species	F (1, 104) = 4022	P<0,0001
Fig 1K, 3K	iWAT weight/final BW (%)	Species x diet x sex	F (1, 150) = 0,2074	P=0,6495
		Species x diet	F (1, 150) = 268,0	P<0,0001
		Species x sex	F (1, 150) = 6,917	P=0,0094
		Diet	F (1, 150) = 462,8	P<0,0001
		Sex	F (1, 150) = 5,659	P=0,0186
		Species	F (1, 150) = 348,1	P<0,0001
Fig 1L, 3L	gWAT weight/final BW (%)	Species x diet x sex	F (1, 150) = 76,08	P<0,0001
		Species x diet	F (1, 150) = 86,65	P<0,0001
		Species x sex	F (1, 150) = 70,09	P<0,0001
		Diet	F (1, 150) = 141,1	P<0,0001
		Sex	F (1, 150) = 38,60	P<0,0001
		Species	F (1, 150) = 497,1	P<0,0001
Fig 2A, 4A	Locomotor activity (counts/30min)	Species x diet x sex	F (1, 148) = 0,0188	P=0,8909
		Species x diet	F (1, 148) = 15,21	P=0,0001
		Species x sex	F (1, 148) = 5,638	P=0,0189
		Diet	F (1, 148) = 22,18	P<0,0001
		Sex	F (1, 148) = 30,60	P<0,0001
		Species	F (1, 148) = 46,01	P<0,0001
Fig 2C, 4C	BAT temperature (°C)	Species x diet x sex	F (1, 151) = 0,0080	P=0,9287
		Species x diet	F (1, 151) = 32,15	P<0,0001
		Species x sex	F (1, 151) = 5,331	P=0,0223
		Diet	F (1, 151) = 0,3061	P=0,5809
		Sex	F (1, 151) = 41,41	P<0,0001
		Species	F (1, 151) = 0,0627	P=0,8025

Supplementary Table S1. Results of 3-way ANOVAs directly comparing key parameters from the rat and mouse datasets. Key interaction of species and diet is highlighted in yellow, and significant for all parameters analyzed.
